# Identifying the ionically bound cell wall and intracellular glycoside hydrolases in late growth stage *Arabidopsis* stems: implications for the genetic engineering of bioenergy crops

**DOI:** 10.3389/fpls.2015.00315

**Published:** 2015-05-13

**Authors:** Hui Wei, Roman Brunecky, Bryon S. Donohoe, Shi-You Ding, Peter N. Ciesielski, Shihui Yang, Melvin P. Tucker, Michael E. Himmel

**Affiliations:** ^1^Biosciences Center, National Renewable Energy LaboratoryGolden, CO, USA; ^2^Department of Plant Biology, Michigan State UniversityEast Lansing, MI, USA; ^3^National Bioenergy Center, National Renewable Energy LaboratoryGolden, CO, USA

**Keywords:** cell wall proteins, glycoside hydrolase, xylan-modifying enzyme, pectin-modifying enzyme, plant late growth stage, plant senescence, biomass recalcitrance, feedstock engineering

## Abstract

Identifying the cell wall-ionically bound glycoside hydrolases (GHs) in *Arabidopsis* stems is important for understanding the regulation of cell wall integrity. For cell wall proteomics studies, the preparation of clean cell wall fractions is a challenge since cell walls constitute an open compartment, which is more likely to contain a mixture of intracellular and extracellular proteins due to cell leakage at the late growth stage. Here, we utilize a CaCl_2_-extraction procedure to isolate non-structural proteins from *Arabidopsis* whole stems, followed by the in-solution and in-gel digestion methods coupled with Nano-LC-MS/MS, bioinformatics and literature analyses. This has led to the identification of 75 proteins identified using the in-solution method and 236 proteins identified by the in-gel method, among which about 10% of proteins predicted to be secreted. Together, eight cell wall proteins, namely AT1G75040, AT5G26000, AT3G57260, AT4G21650, AT3G52960, AT3G49120, AT5G49360, and AT3G14067, were identified by the in-solution method; among them, three were the GHs (AT5G26000, myrosinase 1, GH1; AT3G57260, β-1,3-glucanase 2, GH17; AT5G49360, bifunctional XYL 1/α-L-arabinofuranosidase, GH3). Moreover, four more GHs: AT4G30270 (xyloglucan endotransferase, GH16), AT1G68560 (bifunctional α-l-arabinofuranosidase/XYL, GH31), AT1G12240 (invertase, GH32) and AT2G28470 (β-galactosidase 8, GH35), were identified by the in-gel solution method only. Notably, more than half of above identified GHs are xylan- or hemicellulose-modifying enzymes, and will likely have an impact on cellulose accessibility, which is a critical factor for downstream enzymatic hydrolysis of plant tissues for biofuels production. The implications of these cell wall proteins identified at the late growth stage for the genetic engineering of bioenergy crops are discussed.

## Introduction

Currently, plant cell wall recalcitrance (caused by cell wall integrity and strength) is one of the major hurdles for the development of economically viable biomass-based biofuels (Himmel et al., 2007; Dashtban et al., 2009; Ding et al., 2012; Inoue et al., 2014). Plant cell walls are predominantly composed of cellulose, hemicellulose, and lignin. In addition, they also contain both enzymes and structural proteins. Most plant cell wall proteins go through post-translational modifications, including *N*-glycosylation, *O*-glycosylation, the addition of glycosylphosphatidylinositol (GPI)-anchor, or hydroxylation of proline residues transforming them to hydroxyproline (see review Albenne et al., 2013). Cell wall structural proteins usually account for 1–5% (mass)of cell wall, and can be characterized into three major classes: extensins, hydroxyproline/proline-rich proteins, and glycine rich proteins (Carpita et al., 2001). The other group of cell wall proteins is non-structural, and includes enzymes that contribute a range of functions during growth and development including stress response, oxido-reductase activities, and hydrolytic activities (Carpita et al., 2001; Minic et al., 2007; Jamet et al., 2008). Note that an ideal classification system for cell wall proteins is still lacking, as the above classifications cannot accommodate cell wall proteins like enzyme inhibitors as well as lectins that have no enzymatic activities. For the purpose of exploring potential routes for engineering bioenergy crops, in this study we focus on the non-structural cell wall proteins.

In the past decade, 605 plant cell wall proteins have been annotated in the *Arabidopsis* Information Resource (TAIR) Gene Ontology (GO) cellular component annotation database on a genome-wide basis (as of December 2013). Among them, about 500 of the proteins predicted to be secreted have been recorded in WallProtDB (http://www.polebio.scsv.ups-tlse.fr/WallProtDB/). These proteins are collectively identified by various proteomic studies using different plant tissue materials. As the studies toward a complete cell wall proteome progress, the number of cell wall proteins are likely to exceed the above number (Jamet et al., 2006; Albenne et al., 2013).

Recent proteomic studies have identified numerous glycoside hydrolases (GH) and carbohydrate esterases (CE) from *Arabidopsis* cell walls from different tissues at different developmental stages; these include cell suspension cultures (Robertson et al., 1997; Chivasa et al., 2002; Borderies et al., 2003; Bayer et al., 2006), cell wall-regenerating protoplasts (Kwon et al., 2005), etiolated seedlings (culture medium) (Charmont et al., 2005), 5- to 11-day-old hypocotyls (Feiz et al., 2006),(Irshad et al., 2008; Zhang et al., 2011), roots of 18-day-old seedlings (Basu et al., 2006), leaves of 4- to 5-week-old rosettes (Haslam et al., 2003; Boudart et al., 2005), and plant stems at the late flowering stage (Minic et al., 2007). Note that in this study, the late flowering stage is defined as middle growth stage, which is in consistent with the literature (Minic et al., 2007). However, no studies to date have focused on *Arabidopsis* stems at late pod stage (defined as late growth stage in this study), which are more relevant to the study of harvested biomass used for downstream conversion to biofuels and chemicals. The relevance of also characterizing the total soluble proteins in plant stems is that some soluble proteins could impact—either enhance or impede—the downstream saccharification of biomass and sugar release yield.

There is another gap between the current study of plant cell walls and the need for developing plant biomass-to-biofuels conversion technology. The conventional methods for characterizing plant cell wall proteins usually start with cell wall fractionation (Fry, 1988; Feiz et al., 2006), for which the main problem is that the biomass-to-biofuels conversion process utilizes the whole plant shoot biomass (in which the stems are the major tissue), not the purified cell walls. Accordingly, for the prevailing methods used for characterizing transgenic plants that overexpress cellulolytic genes, the amount of expressed target enzymes are often measured and presented as percent of the total proteins found in the whole plant tissues, such as leaves or stems (Dai et al., 1999), and not the fractionated cell wall. Furthermore, no matter how precise the cell wall purification procedure is, contamination with intracellular proteins accounts for approximately 10% of the total protein (Feiz et al., 2006), and the percentage of intracellular protein contaminants are likely to vary between different plant materials (Albenne et al., 2013, 2014). Identifying these contaminating proteins requires *a priori* knowledge of cell wall protein inventory and bioinformatics analyses. Therefore, we were compelled to use a different procedure based on recent literature; using CaCl_2_ to extract total ionically bound proteins from whole stems (without cell wall fractionation) (Minic et al., 2007), as an initial step for protein sample preparation. This approach increases the relevance of the study to bioenergy crop conversion where the whole stem biomass is used as feedstock.

Researchers at the National Renewable Energy Laboratory, including some authors of this paper, have collaborated for some time with other groups in expressing heterologous cellulases in plants (Dai et al., 1999, 2005; Ziegler et al., 2000; Himmel et al., 2007; Sun et al., 2007; Taylor et al., 2008; Brunecky et al., 2011), and in conducting the chemical, physical, enzymatic, and imaging characterization of native as well as genetic engineered plants (Penning et al., 2009; Ziebell et al., 2010; Brunecky et al., 2012; Bonawitz et al., 2014; Ciesielski et al., 2014; Im Kim et al., 2014; Xiao et al., 2014). We have ongoing research projects expressing various GHs and biocatalysts in plants (Wei et al., 2015), for which we need (1) a better understanding of both the presence and the abundance of endogenous GHs in plant stems, (2) candidate abundant cell wall proteins to act as docking sites for displaying foreign cellulases and xylanases on the cell wall matrix of plant stems via covalent link or protein fusion approaches, and (3) more efficient promoters to control the temporal and spatial expression of target proteins and biocatalysts in plants.

The key objectives of this study are to fill knowledge gaps about the compositions of cell wall proteins including GHs in the context of whole *Arabidopsis* stem proteome at the late growth stage. We believe that understanding more about these hydrolytic stem proteins has the potential for enhancing biomass-to-biofuels technology development. Our assumption here is that during the late growth stage, both the ionically bound cell wall proteins and the leaked-out intracellular proteins are important factors affecting cell wall integrity due to their relatively high mobility (compared to the structural proteins of cell walls and plasma membranes).

To achieve these goals, we used both the in-solution and in-gel digestion methods coupled with LC-MS/MS to identify the soluble proteins from *Arabidopsis* whole stems. The cell wall associated proteins were determined using Gene Ontology (GO) cellular component annotation and secretion signal peptide prediction, coupled with published experimental evidence. In contrast, the GH assignments were based on the CAZy database (http://www.cazy.org/), coupled with published experimental evidence. The potential use of the identified GHs and their promoter sequences for engineering biomass crops with the aim of enhancing deconstruction of plant cell walls is also discussed.

## Materials and methods

### Plant materials

Plants of *Arabidopsis thaliana* ecotype Col-0 were grown in pots in a greenhouse between October and May, under long-day conditions (14 h light/10 h dark) at 23°C. Three independent batches of plants were grown to provide replicates for repeating the protein extractions and subsequent nanoflow liquid chromatography-electrospray ionization-tandem mass spectrometry (Nano-LC-ESI-MS/MS) analysis.

### Transmission electron microscopy

Samples of *Arabidopsis* stem were prepared for transmission electron microscopy (TEM) by high-pressure freezing and freeze substitution according to the literature (Donohoe et al., 2006). Briefly, samples were cryo-preserved using high-pressure freezing in a Leica EM-Pact2 and freeze substituted in 1% OsO_4_ over several days in a Leica AFS2 (Leica, Wetzlar, Germany). Samples were infiltrated with Eponate 812 (EMS, Hatfield, PA) by incubating at room temperature for several h to overnight in increasing concentrations of resin (15, 30, 60, 90%, 3 × 100% resin, diluted in acetone). The samples were transferred to capsules and the resin polymerized in an oven at 60°C overnight. Resin embedded samples were sectioned to approximately 50 nm with a Diatome diamond knife on a Leica EM UTC ultramicrotome (Leica, Wetzlar, Germany). Sections were collected on 0.5% Formvar coated slot grids (SPI Supplies, West Chester, PA). Grids were post-stained for 4 min with 2% aqueous uranyl acetate and for 2 min with Reynold's lead citrate. Images were taken with a 4 mega-pixel Gatan UltraScan 1000 camera (Gatan, Pleasanton, CA) on a FEI Tecnai G2 20 Twin 200 kV LaB6 TEM (FEI, Hillsboro, OR).

### CaCl_2_ extraction of proteins from late growth stage stems

The CaCl_2_ extraction method was chosen because CaCl_2_ has been reported to be the most efficient salt for cell wall protein extraction (Boudart et al., 2005; Feiz et al., 2006; Minic et al., 2007). The stem protein extraction procedure used here was based on previous literature reports (Minic et al., 2007) for extracting cell wall proteins from *Arabidopsis* mature stems. *A. thaliana* Col-0 plants at the late silique stage (i.e., pods beginning to desiccate) were used, from which the top 5-cm segment of the stems, the siliques, and the remaining leaves were removed from the main stems. Approximately 4 g of stems from 10 plants (18–22 cm in length) of *A. thaliana* were pooled and suspended in 6 mL of ice-cold extraction buffer consisting of 25 mM BisTris pH 7.0, 200 mM CaCl_2_, 10% (v/v) glycerol, 4 μM Na-cacodylate, and 1/200 (v/v) protease inhibitor cocktail (P-9599, Sigma Chemical, St Louis, MO). The plant materials were ground in a mortar with a pestle for 5 min. The ground plant material was then centrifuged three times for 3 min at 10,000 × g at 4°C, and the resulting supernatant was further centrifuged at 17,000 × g for 15 min. The final supernatant was collected as the protein extract of the stems, and its total protein concentration was determined with the Micro BCA Protein Assay Kit from Thermo Scientific (Rockford, IL). The protein extracts were stored at -80°C.

### In-solution digestion method for direct identification of stem proteins

This method aimed to provide a direct identification analysis of proteins in the extracted protein solution from stem tissues. The above extracted *Arabidopsis* stem protein solution was shipped on dry ice to ProtTech Inc. (Phoenixville, PA, USA). For the gel electrophoresis, the protein samples were treated with 6M guanidine HCl, DTT and iodoacetemide before mixing with sample loading buffer. SDS-PAGE was performed using NuPage 12% Bis-Tris gel (Life Technologies, USA), was run at a constant voltage of 200 V for 40 min. Then the gel was fixed with 50% methanol 10% acetic acid for 1 h and then stained overnight with 0.1% Coomassie Brilliant Blue with 50% methanol 10% acetic acid. Gel was then destained with distilled water and then with 50% methanol 10% acetic acid until the background is clear.

For the in-solution trypsin digestion of protein sample, the endoproteinase trypsin (modified, sequencing grade) obtained from Promega (Madison, WI). The in-solution trypsin digestion was carried out in 100 mM NH_4_HCO_3_ (pH 8.0) at 37°C for 4 h, with a protein to trypsin ratio of 1:30. The reaction was stopped by adding formic acid to 0.5%. The resultant peptide mixture was further analyzed using a Nano-LC-MS/MS system, as described in the later section of LC-MS/MS AND PROTEOMIC ANALYSIS. Three separate runs of above analyses from prepared plant protein samples were conducted, which allows a statistic analysis for the for the identified stem proteins.

### In-gel digestion method for improving the identification of stem proteins

For reproducibility of experiments, the protein gel band method used two batches of plant samples for protein extraction (as described above) and subsequent analyses. This approach included the following five steps, mainly carried out at ProtTech Inc. (Phoenixville, PA, USA).

1. Protein sample treatment and SDS-PAGE. Protein samples were treated with 6 M guanidine-hydrochloride, 3 mM DTT, and 40 mM iodoacetemide to solubilize the samples before being mixed with sample loading buffer. SDS-PAGE was performed using NuPage 4–12% Bis-Tris Gel (Life Technologies, USA). Samples were electrophoresed at a constant current of 120 mA per gel for 1 h. The gel was then fixed with a solution of 50% methanol/10% acetic acid for one h and then stained overnight with 0.1% Coomassie brilliant blue dissolved in 50% methanol/10% acetic acid. Finally, the gel was destained with distilled water and then with 50% methanol/10% acetic acid until the background was clear.
2. The extracted and quantitated protein sample was fractionated by SDS-PAGE resulting into 20 strong bands (S0–S19) and 25 weak bands (W1–W25, covering the other gel areas after the strong bands were removed); thus a total of 45 bands were obtained (see the Results section for details).
3. Each protein gel band was destained, cleaned, and digested in-gel with sequencing grade modified trypsin, as described in literature (Thangthaeng et al., 2011; Yan et al., 2014).
4. The resultant peptide mixture was analyzed using an LC-MS/MS system, as described below.

### LC-MS/MS and proteomic analysis

The protein identification work was carried out at ProtTech Inc. (Phoenixville, PA, USA) using the Nano-LC-ESI-MS/MS for peptide sequencing, which can identify proteins with an ultra-high sensitivity. The same service with similar HPLC and LC-MS/MS settings has been used successfully in numerous proteomic analyses in literature (Thangthaeng et al., 2011; Abdelmegeed et al., 2013; Yan et al., 2014; Li et al., 2015). Briefly, the above prepared, trypsin-digested peptides mixture was subjected to a LC-MS/MS system, in which HPLC with a reverse phase C18 column (75 μm ID) was in-line coupled to a quadrupole ion trap mass spectrometer (LCQ Deca XP PLUS, Thermo, Palo Alto, CA). The solvent A for HPLC was 97.5% water, 2% acetonitrile, 0.5% formic acid, whereas the solvent B was 9.5% water, 90% acetonitrile, 0.5% formic acid. The gradation time from 2% Solvent B to 90% solvent B was 60 min, plus 20 min for sample loading and 20 min for column washing. The column flow rate was around 800 nL min^−1^. The low energy collision induced dissociation (CID) process was set for the mass spectrometer to acquire MS/ MS data. The mass spectrometric data acquired were used to search the *Arabidopsis* protein database TAIR10 with ProtTech's proprietary software suite. The output from the database search was manually validated and generated a list of identified proteins. Different from MALDI-TOF based peptide mapping, the results generated from LC-MS/MS (tandem MS) are established on independent peptide sequencing (Yan and Forster, 2009). Only the proteins that were identified with two or more peptides were analyzed and discussed.

The mass spectrometry proteomics data have been deposited to the ProteomeXchange Consortium (Vizcaino et al., 2014) via the PRIDE partner repository (http://www.ebi.ac.uk/pride) (Vizcaino et al., 2013) with the dataset identifier PXD001851 for in-solution digestion method coupled with LC-MS/MS, and PXD001852 for in-gel digestion method coupled with LC-MS/MS.

A recent proteomic study of cell walls of alfalfa stems, conducted by Verdonk et al., used spectral counts as a practical, label-free way to quantify individual protein abundance by analyzing the MS/MS data with MaxQuant, thus gave a rough estimate of protein abundance (Verdonk et al., 2012), but the researchers raised caution in interpreting the data due to limited experiments. In this study, the relative abundance for each protein identified was calculated based on another label-free method published by Griffin et al. (2010). However, the complexity and the interpretation of the relative abundance for individual proteins are beyond the scope of this study, thus the presented data and discussion were focused on the peptide hits numbers for individual proteins.

### GO annotation and categorization: identification of possible cell wall proteins

The set of locus identifiers for the identified total protein-encoding genes generated from the above procedure was uploaded into the GO annotation website of TAIR (http://www.arabidopsis.org). The *A. thaliana* genome version TAIR10 was used, which contains 27,416 protein coding genes according to TAIR10 statistics. The proteins were grouped into broad functional categories based on the higher level GO terms in the GO hierarchy. We were interested in the GO Cellular Component, the GO Biological Process and the GO Molecular Function characterization. The list of possible cell wall proteins, with their respective characterizations, was exported and manually checked in the National Center for Biotechnology Information (NCBI) gene database for the accuracy of each gene locus.

### Database and literature analyses to confirm cell wall and intracellular GHs and CEs

The above generated list of possible cell wall proteins from the GO annotation analysis was used to conduct fine identification of cell wall protein. Since the Go annotation analysis output relies heavily on bioinformatics prediction and some experimental evidence (Rhee et al., 2008), only a portion of these GO annotation-identified cell wall proteins have experimental support. Thus, for this study, we chose to focus only on the cell wall proteins (including GHs and CEs) confirmed by experimental evidence, which was based on the literature analysis on published cell wall proteomic studies of *Arabidopsis*. In addition, we conducted a bioinformatic analysis to predict the secretion signal peptide (SP) for each of the identified cell wall proteins, using (1) the SignalP 4.1 program with the setting for eukaryotes (http://www.cbs.dtu.dk/services/SignalP/), and (2) the annotation prediction in UniProtKB (http://www.uniprot.org/). The SP prediction annotation in UniProtKB is based on the predictive tools of Phobius, Predotar, SignalP and TargetP, with at least two methods return a positive signal peptide prediction (http://www.uniprot.org/help/signal), thus it is likely to be a more reliable prediction than that using the SignalP program alone; however, some of the identified cell wall proteins have not been annotated yet for their SP prediction.

To identify the intracellular GHs, first, the list of identified total stem proteins was mapped to the GH dataset of *Arabidopsis* at the CAZy database (http://www.cazy.org/e1.html), which led to a list containing all stem GHs. After teasing out the cell wall GHs (based on the above analyses), the identified possible intracellular GHs were matched to literature reports of experimental evidence supporting their intracellular localization.

### Statistic analysis

The data for the peptide hit numbers of the 75 stem proteins among three biological replicates identified by the in-solution method was imported into SAS JMP Genomics 6.0 (SAS Inc., NC). Briefly, the numbers of peptides identified by LC-MS/MS for Replicates 1, 2, 3 were analyzed for data distribution and then the correlations using the multivariate correlation pairwise estimation method.

## Results

### Microscopic features of *Arabidopsis* stem cross-sections

**Figure 1:**
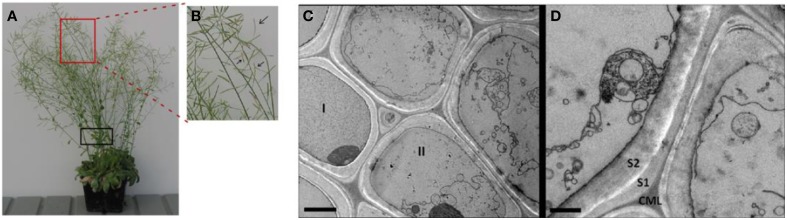
**The *Arabidopsis* plants and the representative transmission electron micrograph (TEM) of cells in the late growth stage *Arabidopsis* stems. (A)** The *Arabidopsis* plants used for CaCl_2_-extraction of plant stem cell walls at late growth stage (i.e., pods beginning to desiccate). **(B)** An enlarged image showing siliques turning color from green to yellow, an indicator that the plant is at the late stage of growth. **(C)** TEM image for sclerenchyma cells of a vascular bundle undergoing senescence. Cell I retains a largely intact cytoplasm, indicating cell is in pre- or very early stages of senescence. Cell II and the others in the field of view are in later stages of senescence, exhibiting deconstructed membranes and organelles. Scale bar 2 μm. **(D)** Membrane and organelle fragments adhere to secondary cell wall surfaces during senescence. Scale bar 1 μm. CML, compound middle lamella; S1, first layer of secondary cell wall; S2, second layer of secondary cell wall. Note that *Arabidopsis* stem segments used for TEM analysis is indicated by black box in **(A)**.

In plants, programmed cell death (PCD) can occur during many abiotic stress conditions and developmental processes, including the tracheary element formation and plant senescent processes (Jabs et al., 1996; Kuriyama and Fukuda, 2002; Gunawardena et al., 2004; Van Breusegem and Dat, 2006). Our microscopic observation of late growth stage *Arabidopsis* stems shows that a significant proportion of plant cells and their organelles were broken, resulting in segments and debris of plasma membranes and organelles in the stems at the late growth stage (Figures 1A–D). The image shows the broken cell membrane, and highlights the *in vivo* cell walls at the late growth stage are already mixed with intracellular components and protein. This is one more rationale for choosing a non-cell wall fractionation approach for extracting both the ionically bound cell wall and intracellular proteins Note that the proportion of intracellular proteins that remain in the senesced cell walls in from *Arabidopsis* stems.

Note that the proportion of intracellular proteins that remain in the senesced cell walls in Figure 1 was not quantified. However, additional microscopy data illustrate differences between cells in the later stage (undergoing senescent; Supplementary Figures S1A,B) and the earlier stage (active growth and flowering; Supplementary Figures S1C,D). Although there was variability among the cells within a tissue section, there are two features that are not seen in earlier growth stages. The first is a cell lumen filled with vesicles and multi-vesicular bodies of various shapes and sizes (black arrows in Supplementary Figure S1A). The second feature is that the cell walls develop a dense staining layer on their lumenal surface (white arrows in Supplementary Figures S1A,B). In other words, we do consistently see evidence for the vesiculation and degradation of cellular organelles and the concomitant development of an electron dense layer on the cell wall lumenal surface that we interpret as being a protein-containing cellular residue (Supplementary Figures S1A,B).

In contrast, the typical stem cells from earlier growth stages display intact cytoplasms that contain recognizable organelles such as chloroplast, mitochondria, ER, Golgi etc., all surrounding an intact central vacuole (Supplemental Figures S1C,D).

### Stem proteins revealed by in-solution digestion method

**Figure 2:**
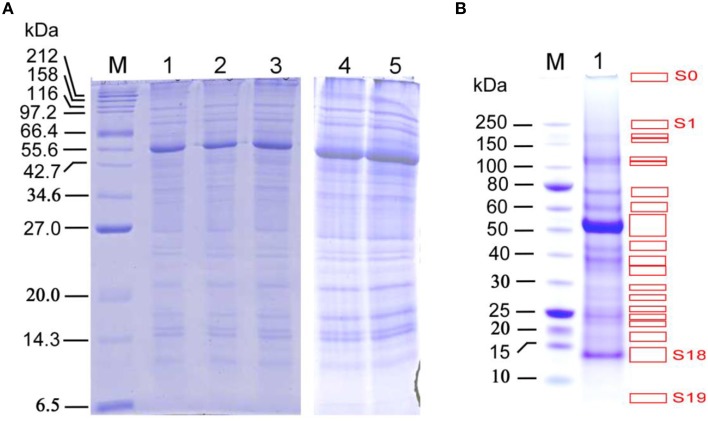
**SDS-PAGE analysis of proteins extracted from the late growth stage *Arabidopsis* stems for the in-solution and in-gel digestion approaches in identifying stem proteins. (A)** SDS-PAGE analysis with a range of protein loading amount, Lanes 1–3, the three biological replicate samples used for in-solution digestion analysis; 10 μg proteins per well. Lanes 4 and 5, the two biological replicate samples used for in-gel digestion analysis; 30 μg proteins per well. M: protein marker (#P7702, New England Biolabs, USA); NuPage 12% Bis-Tris gel was used for better separation of small- to medium-size proteins (15–75 kDa). **(B)** SDS-PAGE separation of proteins used for in-gel digestion analysis, with 30 μg per well. M: protein marker (#P7703, New England Biolabs, USA). NuPage 4–12% Bis-Tris gel was used for better separation of medium- to large-size proteins (30–250 kDa), which resulted in 20 distinguishable strong bands (S0–S19, indicated by red boxes) and 25 weak bands (W1–W25; covering the remaining gel areas after strong bands were removed), which were used for digestion and subsequent LC-MS analysis. Note that the band S0 is the location of protein-loading well.

In-solution digestion method coupled with LC-MS/MS was used for a direct identification of stem proteins in the three biological replicate sample (Figure 2A), using the procedure described in the Materials and Methods. The protein and peptide details for in-solution digestion method coupled with LC-MS/MS experiments can be found in Supplemental Data Sheets 1–3 for the three biological replicates, respectively. The supplemental data sheets provide the information on (1) the scan number of an identified peptide in a LC-MS/MS file, in which if a peptide has been sequenced more than once, it will have more than one scan numbers; (2) the peptide sequence and the peptide mass calculated from each peptide sequence; and (3) the sequence header (limited to 300 characters) for the identified protein present in TAIR 10 database.

Together, these three replicates led to a common list of 75 stem proteins (see Supplemental Table S1) that are being identified with two or more peptides by LC-MS/MS. Not surprisingly, ATCG00490 (ribulose-bisphosphate carboxylase, RuBisCO) on the top of the list for the number of peptide hits. Other approaches, such as the depletion of RuBisCO, need to be explored in the future to identify more stem proteins, especially the less abundant proteins.

**Figure 3:**
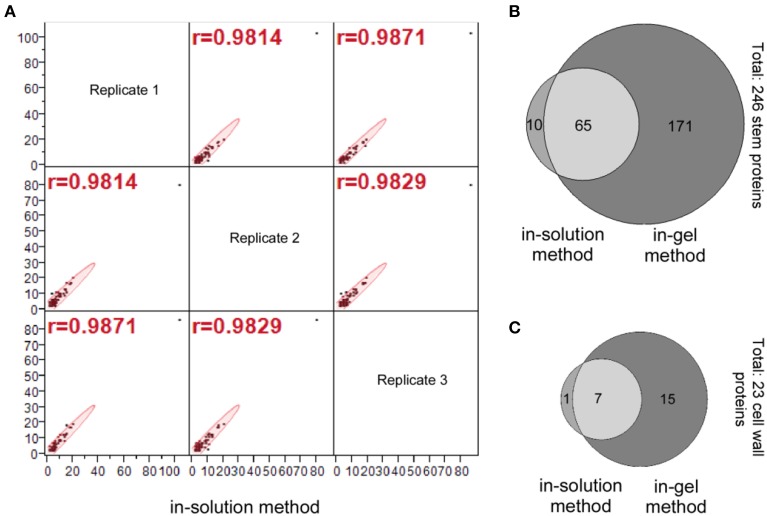
**Identification of the stem proteins by the in-solution and in-gel methods. (A)** Multivariate correlation analysis of the number of peptide hits for the 75 stem proteins that identified by the in-solution method from analyzing the three replicate samples. **(B)** Venn diagram for the number of stem proteins, and **(C)** Venn diagram for the number of cell wall proteins identified by in-solution and in-gel methods coupled with LC-MS/MS analyses, respectively. A comparison of **(B)** vs. **(C)** indicates that the number of cell wall proteins accounts for around 10% of total stem proteins identified both in-solution and in-gel methods, which is a reflection of the fact that soluble proteins are extracted from the whole *Arabidopsis* stems.

Our statistical analysis for the number of peptide hits of the 75 stem proteins (Column D, E, and F; Supplemental Table S1) indicate a high correlation among three replicates generated by the in-solution method; the data distributions of these three replicates are similar with a mean value of 2.3–2.4 for the number of peptide hits per protein, and their correlations are very tight too with the correlation coefficient (*r*-value) greater than 0.98 for all correlations (Figure 3A).

### Cell wall proteins identified by in-solution digestion method

As described in Materials and Methods, the initial identification of possible cell wall proteins is based on the TAIR GO annotation. Briefly, the TAIR locus ID list of identified stem proteins at the late growth stage was put through the TAIR GO annotation, from which the GO annotation Cellular Component output results identified possible cell wall proteins. After the locus ID search of literature published on cell wall proteomic studies of *Arabidopsis*, 8 proteins were confirmed to be the cell wall proteins (as listed in Table 1).

**Table 1:** **List of 8 identified ionically bound cell wall proteins in *Arabidopsis* stems at late growth stage, using the in-solution method coupled with LC-MS/MS**.

**Locus ID**	**Proteins**	**No. of peptides identified**
		**Sample 1, 2, 3**
AT1G75040	Pathogenesis-related gene 5 (PR5)	13, 9, 8
AT5G26000	Myrosinase 1 (thioglucoside glucohydrolase, TGG1); GH1	20, 20, 19
AT3G57260	β-1,3-glucanase 2; GH17	4, 4, 5
AT4G21650	Serine-type protease	9, 8, 8
AT3G52960	Thioredoxin-dependent peroxidase	5, 6, 5
AT3G49120	Peroxidase 34	4, 3, 2
AT5G49360	Bifunctional XYL 1/α-L-arabinofuranosidase; GH3	2, 3, 3
AT3G14067	Subtilase family protein (proteinase inhibitor)	2, 2, 3

*The full list of identified 75 total stem proteins with their relative abundance using protein solution method was provided in Supplemental Table S1. Note that except AT3G14067, all the hereby listed cell wall proteins were also identified by in-gel method as listed Table 2. For AT3G14067, it was identified with 5 and 1 peptides in the biological replicates 1 and 2 analyses using in-gel method, respectively*.

Among them, three were GHs that include GH1 (AT5G26000, myrosinase 1), GH17 (AT3G57260, β-1,3-glucanase 2), and GH3 (AT5G49360, bifunctional XYL 1/α-L-arabinofuranosidase).

### More stem proteins identified by in-gel digestion method

As described in the Materials and Methods, the two biological replicate extracted proteins (Figure 2A; estimated to be approximately 4.1 mg/mL for a representative preparation) from *Arabidopsis* stems collected at late growth stage were used to conduct another round of LC-MS/MS analysis, aiming to identify more stem proteins by using the in-gel digestion method.

To determine the suitable loading of total stem proteins for gel slicing and subsequent analyses, protein samples were loaded into wells over a broad range, from 3 to 192 μg per well, followed by SDS-PAGE and Coomassie brilliant blue staining. The resulting SDS-PAGE images show that a loading amount of 30 μg per well allows the best separation for both strong and weak protein bands (Figure 2B), In contrast, if the mixed protein extract was used without prior SDS-PAGE separation and then subjected to protein identification via above in-solution digestion approach, then the proteins with low abundance may have been missed.

The 20 strong (S0–S19) and 25 weak (W1–W25) protein bands in the SDS-PAGE gel (Figure 2B) were used to conduct in-gel digestion followed by LC-MS/MS analyses, which led to the identification of more stem proteins at late growth stage. The protein and peptide details for each of the 20 strong and 25 weak bands can be found in Supplemental Data Sheets 4, 5 for the two biological replicates, respectively. Analyses of Replicates 1 and 2 identified 629 and 606 proteins with at least one peptide being sequenced. After increasing the filter threshold for the number of peptides being identified to two, both replicates resulted in the identification of 413 proteins, respectively. Together, these two replicates led to a common list of 236 stem proteins (see Supplemental Table S2) that being identified with 2 or more peptides by LC-MS/MS.

A Venn diagram comparing number of identified stem proteins by both in-solution and in-gel methods reveal that 65 proteins were identified by both methods, while 10 proteins identified by in-solution method only and 171 proteins identified by in-gel method only (Figure 3B; also see Supplemental Table S3). It should be noted that based on other reports in the literature it is not unusual that a small proportion of plant proteins identified by in-solution method were not identified by the in-gel method (Lundquist et al., 2012). The fact that some proteins were identified with the in-gel method but not by the in-solution method suggests that the separation of peptides by the LC might not be efficient enough, and the tryptic digestion might be more efficient on the in-gel denatured proteins.

In summary, the aforementioned data demonstrated that the in-gel digestion method detected three times more stem proteins as well as identifying more cell wall proteins than the in-solution method (Figures 3B,C).

It is noteworthy that the reproducibility between the two replicates of SDS-PAGE fractionation and in-gel digestion approach seems to be affected by the fact that the gel cut was conducted manually by using razors, making it challenging to obtain the same gel bands between the two replicates, thus causing variation between two replicates for the less abundant proteins identification in a specific gel band. In addition, the aforementioned data showed that 413/629 to 413/606 (i.e., 66–68%) of identified proteins have two or more specific proteins being sequenced by LC-MS/MS. For future studies, to increase the accuracy and effectiveness in identifying the remaining 22–24% proteins with only one peptide being sequenced by LC-MS/MS, we suggest two possible improvements:

1. For strong protein gel bands, each band might still contain many different proteins, which can still impede the identification of low abundant proteins contained in that band. Ideally, this type of strong protein gel bands should be cut further into thin parallel bands for the LC-MS/MS analysis, if the cost for experiments is not an issue.
2. For weak protein gel bands, we speculate that more protein loading amount per well will increase the protein contents in each of the 25 weak bands in Figure 2B, thus will likely improve the identification of the low abundant proteins in each weak protein gel band.

These two improvements will likely identify more proteins, thus improve the reproducibility between the replicates of in-gel digestion approach.

### GO functional analysis of identified stem proteins

**Figure 4:**
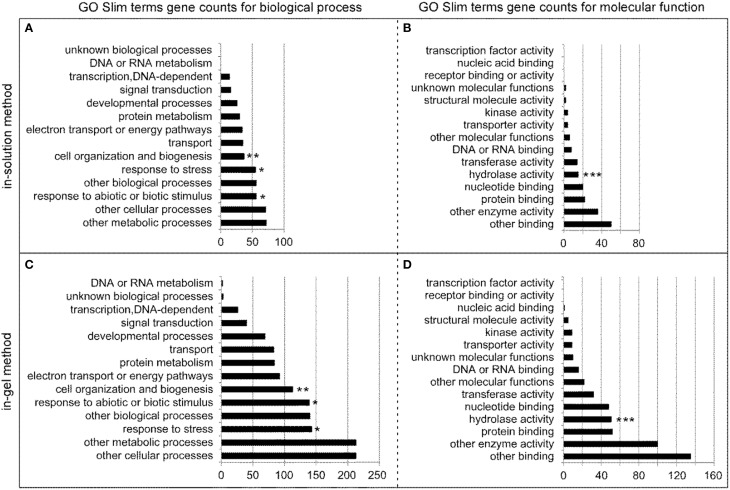
**GO Slim annotations for the biological process and molecular function of the 75 and 236 relatively abundant stem proteins identified by in-solution and in-gel methods, respectively**. Annotation gene counts for the biological process **(A)** and molecular function **(B)** of stem proteins identified by the in-solution method are illustrated in the upper two panels, whereas those for the biological process **(C)** and molecular function **(D)** of stem proteins identified by the in-gel method are illustrated in the lower two panels. Note that since GO annotation categories overlap, the sum of GO annotated genes is larger than the number of total genes analyzed. The symbols of ^*^, ^**^, and ^***^ are added in the panels to highlight the specific biologic process or molecular function, as described in details in the Results section.

By using the Gene Ontology (GO) terms available at TAIR (www.arabidopsis.org), we performed GO analysis using the 75 and 236 relatively abundant stem proteins identified by in-solution and in-gel methods, respectively. Figure 4 shows the number of genes in the biological process and molecular function categories. For the biological process categories, the “response to stress” and “response to abiotic or biotic stimulus” (marked by ^*^ symbols), and “cell organization and biogenesis” (marked by ^**^) were well-represented among the clearly defined terms for the stem proteins generated by both in-solution (Figure 4A) and in-gel methods (Figure 4C). For the molecular function categories, the “hydrolase activity” (marked by ^***^) was well-represented too (Figures 4B,D), which is closely related to the theme of this study.

### Major components of cell wall proteome and their biochemical activity

The above 236 in-gel digestion method identified stem proteins were subjected to GO annotation Cellular Component analysis, and to the locus ID-based search of literature published on cell wall proteomes of *Arabidopsis*. From the above analysis and search, 22 proteins were confirmed to cell wall proteins, and were classified into functional groups according to the established classification system (Jamet et al., 2006; Minic et al., 2007), as listed in Table 2.

**Table 2:** **List of 22 identified ionically bound cell wall proteins by the in-gel method coupled with LC-MS/MS in stems of *Arabidopsis* at the late growth stage**.

**Locus IDs**	**Proteins (putative or function confirmed)**	**CBM, GH or CE family**	**SP**	
			**SignalP**	**UniprotKB**
**GHs (7)**				
AT5G26000	Myrosinase 1 (thioglucoside glucohydrolase, TGG1)	GH1	1–19	1–19
^*^AT5G49360	Bifunctional XYL 1/α-L-arabinofuranosidase	GH3	1–30	1–30
^*^AT4G30270	Xyloglucan endotransferase	GH16	1–21	1–21
AT3G57260	β-1,3-glucanase 2	GH17	1–30	1–22
^*^AT1G68560	Bifunctional α-l-arabinofuranosidase/XYL	GH31	1–27	1–27
AT1G12240	β-fructofuranosidase (i.e., invertase)	GH32	None	1–67
^*^,^^^ AT2G28470	β-galactosidase 8	GH35	1–23	1–29
**CEs (3)**				
^^^ AT1G11580	Pectin methylesterase (PME)	CE8	None	1–34
^^^ AT3G14310	Pectin methylesterase 3 (PME3)	CE8	None	1–35
AT1G29670	GDSL esterase/lipase		1–24	1–24
**OXIDO-REDUCTASE INCLUDING PEROXIDASE (3)**				
AT3G49120	Peroxidase 34		1–30	1–30
AT3G52960	Thioredoxin-dependent peroxidase		None	None
AT1G76160	Sks5 (SKU5 Similar 5); copper ion binding/oxidoreductase		1–23	n/a
**PROTEASES AND PROTEASES INHIBITORS (3)**				
AT1G47128	Cysteine proteinase/dehydration stress-responsive gene (RD21)		1–21	1–21
AT3G14067	Subtilase family protein (proteinase inhibitor)		1–25	n/a
AT4G21650	Serine-type protease		1–21	n/a
AT4G26690	MRH5 (glycerophosphoryl diester phosphodiesterase)		1–27	1–27
**PROTEINS WITH PROTEIN-PROTEIN INTERACTION DOMAINS (3)**				
AT1G33590	Leucine-rich repeat (LRR) protein		none	n/a
AT1G53070	Legume lectin family protein		1–23	1–23
AT1G78830	Curculin-like (mannose-binding) lectin family protein		1–22	n/a
**STRESS RESPONSIVE AND DEFENSE PROTEINS (2)**				
AT2G45470	Fasciclin-like arabinogalactan protein 8 (FLA8)		1–25	1–25
AT1G75040	Pathogenesis-related gene 5 (PR5)		1–23	1–23
**MISCELLANEOUS (1)**				
AT4G27520	Early nodulin-like protein 2 (ENODL2)		1–28	1–28

The protein categories are arranged by the number of proteins identified in each category. Proteins acting on xylan and pectin are indicated by ^*^ and ^^^ symbols, respectively. Note that the full list of identified 236 total stem proteins using the protein gel method was provided in Supplemental Table S2. Note that for AT3G14067, it was formally counted as one of the 22 identified ionically bound cell wall proteins by the in-gel method; however, because of its being identified with five and one peptides in the two biological replicate analyses of the in-gel method, respectively, as well as its being identified by in-solution method (see Table 1), thus still included in this table. The signal peptides (SP) for the proteins were predicted by using the SignalP 4.1 program and the annotation in UniProtKB, as described in the Materials and Methods as well as the Results sections. none, predicted to be without SP; n/a, the prediction annotation for SP is not available.

As described in the Materials and Methods section, we used the Signal P 4.1 program and the protein annotation in UniProtKB to predict the secretion SP for each of identified cell wall proteins, and the results are presented in Table 2 (last column). All 10 GHs and CEs have positive signal SP predictions in UniProtKB, which are based on the application of a set of predictive tools (see the Materials and Methods section). However, there were two listed proteins in Table 2 (AT3G52960, thioredoxin-dependent peroxidase; AT1G33590, leucine-rich repeat protein) do not have predicted SP by either of the approaches. Since these two proteins are categorized as cell wall proteins in previous literature (see Supplemental Table S4), they are still put as “cell wall proteins,” and future studies are needed to clarify their sub-cellular location.

The biochemical activities of identified GHs and CEs are described below based on their relevance to cell wall structural modification and digestibility:

#### Xylan- or hemicellulose-modifying enzymes

Among the identified cell wall GHs, 4 are GHs that act on xylan or hemicellulose, accounting for more than half of our subset of 7 GHs. These xylan- or hemicellulose-modifying GHs (some with limited experimental evidence) are indicated by ^*^ symbol in Table 2.

The first type is β-galactosidases (AT2G28470, GH35; EC 3.2.1.23) that can remove the galactose side chains attached to arabinose or xylose in arabinoxylan (Ebringerova et al., 1990; De Vries et al., 1999). The roles of this type of enzyme in the regulation of cell wall integrity are supported by reports that the β-galactosidases in mango, papaya, and orange plants were found to be involved in cell wall hydrolysis, as well as fruit ripening and abscission (Ali et al., 1995; Lazan et al., 2004; Wu and Burns, 2004).

The second type of xylan-modifying enzymes herein identified are mainly the two β-D-xylosidases (XYLs; EC 3.2.1.37) that catalyze the hydrolysis of 1,4-β-D-xylo-oligosaccharides and removes successive D-xylose residues from non-reducing ends (Table 2). These XYLs include GH3 (XYL1/α-L-arabinofuranosidase, AT5G49360) and GH31 (α-L-arabinofuranosidase/XYL, AT1G68560. The gene of AT5G49360 (XYL1/α-L-arabinofuranosidase) is expressed specifically in tissues undergoing secondary cell wall thickening (Arsovski et al., 2009). The literature has also shown that some isolated cell wall GHs can act on multiple natural polysaccharides (Sampedro et al., 2001; Lee et al., 2003; Minic et al., 2004), and it has been proposed that this phenomenon of bifunctionality allows efficient modification of cell wall polysaccharides with a limited number of enzymes (Minic et al., 2004, 2006).

The third type is the one xyloglucan endotransferase (XET, AT4G30270, GH16; Table 2). Xyloglucan is the main hemicellulose of primary cell walls in dicots and approximately half of the monocots (Baumann, 2007). XETs can cleave and rejoin xyloglucan chains, characteristics thought to enable two adjacent fibrils to twist against each other while maintaining the cell wall structure (Teleman, 2009).

#### Pectin-modifying GHs and CEs

Among the identified cell wall proteins, there are one GHs and two CEs that act on pectin, as indicated by ^^^ symbol in Table 2. Note that among them, the β-galactosidase (AT2G28470, GH35; Table 2) can modify both xylan and pectin, as galactose is present in both xylan and pectin as side chains (Ebringerova et al., 1990; O'Neill et al., 1990; De Vries et al., 1999).

In addition, two pectin methylesterases (AT1G11580 and AT3G14310, CE8; Table 2) that catalyze the demethylesterification of cell wall polygalacturonans (pectin) into pectate and methanol, were also been detected; these pectin-modifying enzymes are the major determinators that regulate both the degrees of substitution (methylesterification and/or acetylation) and polymerization of pectin, and are involved in the regulation of cell wall integrity and development (Senechal et al., 2014); and thus are worthy further studies.

#### Biochemical activity of xylan- and pectin-modifying enzymes in literature

Although measuring the biochemical activity of identified cell wall GHs and CEs is out of the scope of this study, further literature analysis was conducted for the reported protein purification and functional characterization of some identified cell wall GHs or their homologs. These results could provide additional evidence for the roles of these proteins in regulating cell wall integrity and carbon metabolism.

For gene locus ID AT2G28470, the respective *Arabidopsis* β-galactosidase 8 protein is described above as modifying both xylan and pectin. This protein has been studied experimentally and its rice homolog had been shown to have activity against both xyloglucan and galactans (Kaneko and Kobayashi, 2003).

For gene locus ID AT5G49360 (XYL1, GH3), the encoded protein has been purified from *Arabidopsis* stems and were functionally characterized, confirming their β-D-xylosidase activities (Minic et al., 2004).

It is noteworthy that more studies are needed to confirm the biochemical activity of above 6 identified xylan-, hemicellulose- and pectin-modifying enzymes, especially at the late growth stage.

## Discussion

### Comparison with published cell wall proteome from various tissues and growth stages

#### Size of the identified cell wall proteome

As we mention in the Introduction, numerous proteomic studies have identified cell wall GH and CE enzymes from different *Arabidopsis* cell walls at different developmental stages. Among these, the reported study of the *Arabidopsis* stems at the middle growth stage (Minic et al., 2007) is most comparable to our study in terms of the same tissue type, a partially similar procedures that target ionically bound proteins, and the closeness in growth stages. Minic et al. used CaCl_2_ extraction followed by ConA (Concanavalin A) affinity chromatography to trap and enrich the cell wall protein (Minic et al., 2007), because, most plant cell wall proteins are *N*-glycosylated (Cassab, 1998; Rayon et al., 1998; Minic et al., 2007). It is noteworthy that the number of cell wall GH proteins (which is seven; Table 2) identified in this study is smaller than the number of cell wall GH proteins identified in the middle growth stage *Arabidopsis* stems (which is 30) (Minic et al., 2007), suggesting the effectiveness of the ConA affinity approach in trapping and enriching cell wall proteins.

However, due to the likely difference among individual cell wall proteins for their degree of *N*-glycosylation and affinity to the ConA, their relative abundance is likely to be altered by the ConA enrichment purification, thus cannot be directly used to achieve the objectives in this study for identifying the relative abundant cell wall proteins. Future studies of cell wall proteomes using the ConA affinity approach to trap *N*-glycoproteins or other approaches to prepare cell wall fractions will likely lead to the identification of more late growth stage-associated cell wall proteins.

#### The match of individual cell wall proteins between this study and published cell wall proteome

There have been several cell wall proteomes published in the past decade. Note that due to the fact that different research groups used different procedures in extracting and identifying the cell wall proteins, plus the fact that different *Arabidopsis* tissues or cells at different growth stages were studied, it is challenging to conduct a vis-a-vis comparison between the cell wall proteome of this study and those in literature. Nevertheless, an overall comparison, as illustrated in Supplemental Table S4, reveals that the cell wall proteome of this study has a relatively higher match with *Arabidopsis* stems at the middle growth stage (Minic et al., 2007) and 11-day-old hypocotyls (Feiz et al., 2006).

The above relatively high match with *Arabidopsis* stems at the middle growth stage (Minic et al., 2007) and 11-day-old hypocotyls (Feiz et al., 2006) is consistent with a recently reported large overlap between the proteomes of alfalfa apical and basal stems that represent two developmental stages (Verdonk et al., 2012). In addition, in *Brachypodium distachyon*—a model system for temperate grasses as well as for biofuel crops, 63 cell wall proteins were found to be shared by two organs (i.e., leaves and culms—hollow stems) at two developmental stages (i.e., active growth and mature stage), and were suggested to play housekeeping functions (Douche et al., 2013).

In contrast, the match rate between the stem cell wall proteome of this study and the cell wall proteomes of other tissue types provides a mixed picture. While our stem cell wall proteome has a relatively high match (11 out of 23 identified proteins in this study) with that of 4- to 5-week-old leaves (Jaafar and Zueco, 2004), the match with the 18-day-old seedling root (Basu et al., 2006) and 3-week-old cell suspension culture (Borner et al., 2003) was the lowest, only two or three out of 23 identified proteins in this study being matched, respectively (Supplemental Table S4). These contrasting data prompt us to propose that micro-environmental aeration condition for the plant tissues can affect the composition of their cell wall proteomes. Both *Arabidopsis* stem and leaf tissues are exposed to atmospheric conditions, thus have higher match for their cell wall proteins; in contrast, the plant roots and cell suspension culture are exposed to an underground (for root) or submerged environment with reduced aeration, and with none or subdued light. [The culture condition for 3-week-old cell suspension culture was rotated at 110 rpm at 25°C in subdued light (Borner et al., 2003)]. Future studies are needed to test the above proposal.

### Intracellular GHs: potential for utilizing both soluble and lignocellulosic sugars in bioenergy crops

Thus, far, we have examined the ionically bound cell wall GHs. However, another potentially interesting and important pool of proteins that are often overlooked are the GHs existing in the intracellular compartments of plant cells. From the point of view of biomass conversion, these intracellular GHs could be utilized for downstream enzymatic hydrolysis of biomass. The intracellular GHs were identified using the procedure described earlier, which enabled the identification of three confirmed or likely intracellular GHs (Table 3), as discussed below:

**Table 3:** **List of four identified CaCl_2_-extracted intracellular glycoside hydrolases in stems of *Arabidopsis* at the late growth stage**.

**Locus ID**	**Proteins**	**GH family**	**Subcellular location**	**Literature**
AT1G52400	Glucosidase (Bglu18)	GH1	(C)	Xu et al., 2004
AT5G25980	Myrosinase 2 (thioglucoside glucohydrolase, TGG2)	GH1	V	Xue et al., 1995; Barth and Jander, 2006
AT1G09010	Endo-β-mannosidase	GH2	V	Carter et al., 2004; Jarno, 2011
AT4G15210	β-amylase (BAM5)	GH14	C	Hara et al., 2009; Lisso et al., 2013

*Subcellular locations: C, cytosol; V, vacuole. Those in parentheses indicate uncertainty or the prediction solely based on the gene sequence. Those subcellular location assignments not in parentheses are supported by experimental evidence*.

Two intracellular GH1s were identified in the late growth stage *Arabidopsis* stems, and one of which is β-glucosidase (Bglu18, gene locus ID AT1G52400; Table 3), which was reported to be required in wound-inducible ER body formation and involved in defense/stress responses (Xu et al., 2004). Another intracellular GH1 identified is a myrosinase 2 (AT5G25980, also known as thioglucoside glucohydrolase 2, TGG2), was reported to be involved in the glucosinolate catabolic process by catalyzing the hydrolysis of glucosinolates into compounds that are toxic to various microbes and herbivores, and is thus related to plant defense (Xue et al., 1995; Barth and Jander, 2006). To prevent inappropriate glucosinolate hydrolysis that could generate cytotoxic molecules, the plants need to correctly localize TGG2 in tonoplast (i.e., away from the glucosinolates that likely exist in a different compartment of the cells).

### Implication for the genetic engineering of bioenergy crops

The implication of the identified stem and cell wall proteins in this study for the genetic engineering of bioenergy crops can be elucidated in three aspects: First, in the identified stem proteome, although there are relative abundant xylan- and pectin-acting enzymes, there is lack of major cellulose-degrading enzymes, such as endo-glucanases or exo-cellulases, in the *Arabidopsis* stems at the late growth stage (Table 2). This observation confirms the necessity for expressing exogenous (i.e., bacterial *Acidothermus cellulolyticus* endoglucanase E1 and fungal *Trichoderma reesei* exo-cellobiohydrolase I) in plant cell walls to enhance biomass digestibility (Dai et al., 1999, 2005; Biswas et al., 2006; Sun et al., 2007; Brunecky et al., 2011).

Secondly, in microorganisms the *in vivo* displaying of enzymes via covalently-linkages or fusion to cell wall proteins has been an attractive strategy for using enzymes as catalysts in industrial transformations (Breinig and Schmitt, 2002; Jaafar and Zueco, 2004; Duquesne et al., 2014). The identified relatively abundant cell wall proteins in this study can provide a foundation for applying similar strategy in plants for enhancing the abundance of displayed foreign enzymes by covalently-linked or fused to these cell wall proteins.

Thirdly, as illustrated in Supplemental Table S4, half of the identified cell wall proteins at the late growth stage by this study were also detected in *Arabidopsis* cell wall proteome at the middle stage (i.e., late flowering) in literature. This prompts us to propose that the sequences of their promoters and secretion signals should be explored in future studies for the temporal and spatial control of heterologous cellulase expression, aiming to engineer more digestible biomass crops. The rationale (and assumption) supporting this proposal is that the duration of the heterologous cellulase expression time will have a “cumulative modification effect” on the cellulose of the cell walls throughout both the middle and late stages of plant growth.

## Conclusion

The current study conducted a direct proteomics investigation in identifying the soluble stem proteins among which about 10% of proteins predicted to be secreted. We described both in-solution and in-gel digestion in-solution digestion approaches coupled with LC-MS/MS analyses for identifying *Arabidopsis* stem cell wall proteins at the late growth stage. Although some proteins presented in this study have been identified previously in other tissues and/or at other developmental stages, the confirmation of their presence at the late growth stage indicates that these candidate proteins are more likely to be “carried” to the downstream step and to be utilized more efficiently for the saccharification of biomass.

## Author contributions

MH lead the project, coordinated the study and guided the manuscript preparation. HW designed and executed the experiments, analyzed the data, and prepared the manuscript draft. BD and PC carried out the TEM experiments. RB, BD, SD, SY, and MT contributed to the data analyses and the manuscript preparation. All authors read and approved the final manuscript.

### Conflict of interest statement

The authors declare that the research was conducted in the absence of any commercial or financial relationships that could be construed as a potential conflict of interest.

## Abbreviations

CAZy: Carbohydrate-Active enZYme
CE: carbohydrate esterases
GH: glycoside hydrolases
GO: gene ontology
Nano-LC-ESI-MS/MS: nanoflow liquid chromatography-electrospray ionization-tandem mass spectrometry
ROS: reactive oxygen species
TAIR: The Arabidopsis Information Resource
XYL: β-D-xylosidase.
