# Supplementary material for: Identifying the ionically bound cell wall and intracellular glycoside hydrolases in late growth stage Arabidopsis stems: implications for the genetic engineering of bioenergy crops
Source: Front Plant Sci. 2015 May 13;6:315. doi: 10.3389/fpls.2015.00315 (PMC4429552; doi:10.3389/fpls.2015.00315)
Supplement: Supplementary file 4 [file Table4.DOCX]

**Supplemental Table S4.** The literature match for the 23 cell wall proteins identified in this study showing the detection of these proteins in other tissues and developmental stages of *Arabidopsis*. The table is sorted in the alphabetic order of locus IDs.

| **Locus ID** | **Tissue types, growth stages and literature** | | | | | | | |
| --- | --- | --- | --- | --- | --- | --- | --- | --- |
|  | **stems** | **etiolated hypocotyls** | | | **etiolated seedlings (culture medium)** | **roots** | **leaves** | **cell suspension culture** |
|  | Middle stage (late flowering) | 5 days | 11 days | 11 days | 14 days | 18 day-old seedlings | 4-5 weeks | 3 weeks |
|  | Minic et al., 2007 | Irshad et al., 2008 | Feiz et al., 2006 | Zhang et al., 2011 | Charmont et al., 2005 | Basu et al., 2006 | Boudart et al., 2005 | Borner et al., 2003 |
| AT1G11580 |  | + | + |  |  |  |  |  |
| AT1G12240 | + |  |  |  |  |  |  |  |
| AT1G29670 |  | + | + |  |  |  | + |  |
| AT1G33590 | + | + | + | + |  |  | + |  |
| AT1G47128 |  | + | + |  | + |  | + |  |
| AT1G53070 |  | + |  | + |  |  | + |  |
| AT1G68560 | + | + | + | + |  |  | + |  |
| AT1G75040 |  |  |  |  | + | + | + |  |
| AT1G76160 | + |  | + |  |  |  | + |  |
| AT1G78830 |  | + | + | + |  |  | + |  |
| AT2G28470 |  |  |  |  |  |  | + |  |
| AT2G45470 | + |  |  |  |  |  |  | + |
| AT3G14067 | + |  |  |  |  |  |  |  |
| AT3G14310 |  | + | + | + |  |  | + |  |
| AT3G49120 | + | + | + | + | + |  |  |  |
| AT3G52960 | + |  |  |  |  |  |  |  |
| AT3G57260 |  |  |  |  |  | + | + |  |
| AT4G21650 | + |  |  |  |  |  |  |  |
| AT4G26690 | + |  |  |  |  |  |  | + |
| AT4G27520 |  |  |  |  |  |  |  | + |
| AT4G30270 | + |  | + |  |  |  |  |  |
| AT5G26000 | + |  |  |  |  |  |  |  |
| AT5G49360 | + |  | + |  |  |  |  |  |

Basu, U., Francis, J.L., Whittal, R.M., Stephens, J.L., Wang, Y., Zaiane, O.R., Goebel, R., Muench, D.G., Good, A.G., and Taylor, G.J. (2006). Extracellular proteomes of Arabidopsis thaliana and Brassica napus roots: analysis and comparison by MudPIT and LC-MS/MS. Plant and Soil 286, 357-376.

Borner, G.H., Lilley, K.S., Stevens, T.J., and Dupree, P. (2003). Identification of glycosylphosphatidylinositol-anchored proteins in Arabidopsis. A proteomic and genomic analysis. Plant Physiology 132, 568-577.

Boudart, G., Jamet, E., Rossignol, M., Lafitte, C., Borderies, G., Jauneau, A., Esquerré-Tugayé, M., and Pont-Lezica, R. (2005). Cell wall proteins in apoplastic fluids of Arabidopsis thaliana rosettes: identification by mass spectrometry and bioinformatics. Proteomics 5, 212-221.

Charmont, S.P., Jamet, E., Pont-Lezica, R., and Canut, H. (2005). Proteomic analysis of secreted proteins from Arabidopsis thaliana seedlings: improved recovery following removal of phenolic compounds. Phytochemistry 66, 453-461.

Feiz, L., Irshad, M., Pont-Lezica, R., Canut, H., and Jamet, E. (2006). Evaluation of cell wall preparations for proteomics: a new procedure for purifying cell walls from Arabidopsis hypocotyls. Plant Methods 2, 10.

Irshad, M., Canut, H., Borderies, G.L., Pont-Lezica, R., and Jamet, E. (2008). A new picture of cell wall protein dynamics in elongating cells of Arabidopsis thaliana: confirmed actors and newcomers. BMC Plant Biology 8, 94.

Minic, Z., Jamet, E., Négroni, L., Arsene Der Garabedian, P., Zivy, M., and Jouanin, L. (2007). A sub-proteome of Arabidopsis thaliana mature stems trapped on Concanavalin A is enriched in cell wall glycoside hydrolases. Journal of Experimental Botany 58, 2503-2512.

Zhang, Y., Giboulot, A., Zivy, M., Valot, B., Jamet, E., and Albenne, C. (2011). Combining various strategies to increase the coverage of the plant cell wall glycoproteome. Phytochemistry 72, 1109-1123.
