# Supplementary material for: Identifying the ionically bound cell wall and intracellular glycoside hydrolases in late growth stage Arabidopsis stems: implications for the genetic engineering of bioenergy crops
Source: Front Plant Sci. 2015 May 13;6:315. doi: 10.3389/fpls.2015.00315 (PMC4429552; doi:10.3389/fpls.2015.00315)
Supplement: Supplementary file 10 [file Image1.PDF]

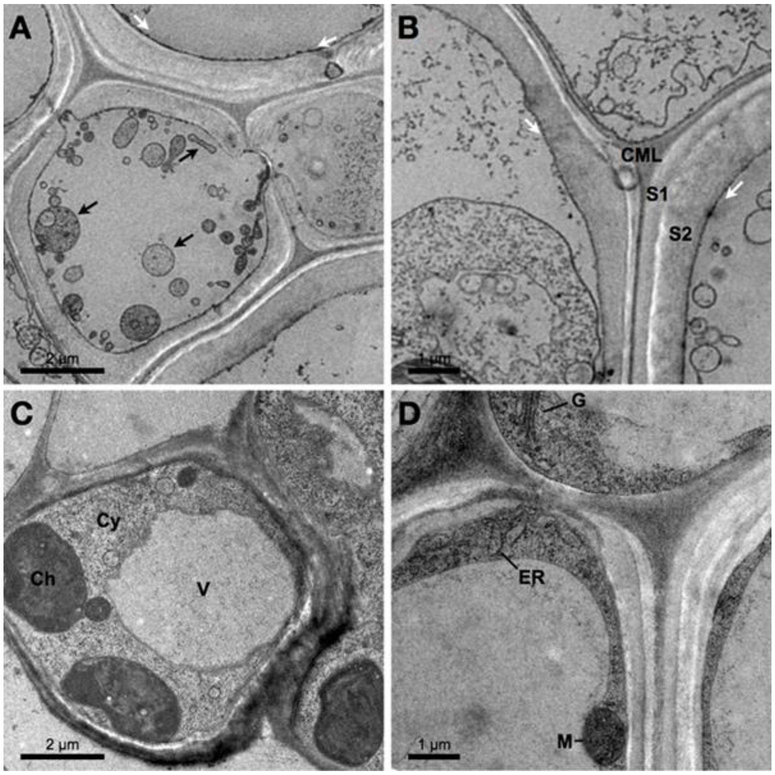

Supplemental FIGURE S1. Additional TEM micrographs of *Arabidopsis* stem cells at the late versus earlier growth stages. **(A, B)** Cells from the late stage plants exhibiting signs of undergoing senescence. **(C, D)** Cells from plants in earlier growth stages including these middle stage (early flowering) sclerenchyma cells display intact cytoplasm (Cy) surrounding a central vacuole (V). Other organelles including chloroplast (Ch), mitochondria (M), endoplasmic reticulum (ER) and Golgi (G) are also seen intact within the cytoplasm pressed against the cell walls. CML, compound middle lamella; S1, first layer of secondary cell wall; S2, second layer of secondary cell wall.
